# Protective Factors Against Social Exclusion in Adolescents: Physical Condition and Physical Activity

**DOI:** 10.3390/children12050635

**Published:** 2025-05-15

**Authors:** Josune Rodríguez-Negro, Javier Murillo-Moraño, Ángel Garrido, Antonio J. Rodríguez-Hidalgo, Juan de Dios Benítez-Sillero

**Affiliations:** 1 Department of Physical Education and Sport, Faculty of Education and Sport, University of the Basque Country (UPV/EHU), 01007 Vitoria-Gasteiz, Spain; 2 Research Group (GIKAFIT), University of the Basque Country (UPV/EHU), 01007 Vitoria-Gasteiz, Spain; 3 Teacher Training College “Sagrado Corazón”, University of Cordoba, 14006 Cordoba, Spain; 4 Research Group in Sport and Physical Education for Personal and Social Development (GIDEPSO), University of Córdoba, 14071 Cordoba, Spain; 5 Department of Physical Education and Sports, University of Granada, 18071 Granada, Spain; 6 Department of Psychology, University of Córdoba, 14071 Cordoba, Spain; 7 Department of Specific Didactics, University of Córdoba, 14071 Cordoba, Spain

**Keywords:** social exclusion, EUROFIT, adolescents

## Abstract

**Background:** Social exclusion in children and adolescents can lead to negative effects such as anxiety, low self-esteem, and academic difficulties. Physical activity and good physical condition could act as protective factors by promoting social integration and emotional well-being. **Methods:** The aim of this study was to analyse whether participation in physical activity and good physical fitness test protect against social exclusion in adolescents aged 12 to 19. A cross-sectional descriptive study was conducted with 876 adolescents, assessing physical activity, physical fitness test (EUROFIT), BMI, and social exclusion (both subtle and manifest) through questionnaires and physical tests. Spearman correlations, ANOVA, and stepwise regression analysis were applied. **Results:** Adolescents who participated in organised physical activities, such as team sports, opposition, or contact sports, showed lower levels of both manifest and subtle exclusion. Aerobic endurance, age, abdominal strength, and gender were predictors of manifest exclusion, while aerobic endurance, age, and participation in organised physical activities predicted subtle exclusion. **Conclusions:** Physical condition and participation in physical activity protect against social exclusion. Taking part in competitive team activities involving contact and opposition is associated with lower levels of exclusion. It is essential to promote strategies that enhance inclusion and well-being among young people.

## 1. Introduction

Social exclusion among peers during childhood and adolescence is a complex phenomenon. It is a process by which minors are marginalised or excluded from activities and opportunities that should be natural and legitimate within their relational context, due to the actions or omissions of other peers [1,2]. Social exclusion has negative effects on its victims, such as low self-esteem, loneliness, anxiety, lack of motivation, poor academic performance, school dropout, increased aggressive behaviour, internalisation or externalisation of other issues, reduced emotional well-being, and more serious disorders such as depression or even suicide [3,4,5,6,7,8]. For this reason, social exclusion has been recognised as a public health issue [9,10].

Unfortunately, social exclusion is still, on many occasions, not recognised as an intentional form of harm. For example, when it is less apparent, occurs without direct confrontation, and leads to situations of isolation [2,11]. Although it may not involve explicit episodes of rejection, it is nonetheless painful for its victims, as it causes emotional and mental harm, potentially triggering a traumatic process with serious consequences [12,13].

Scientific literature in recent years [4,14,15,16,17] has provided evidence pointing to the existence of two subtypes of social exclusion among peers:

(a) one generally referred to as rejection, characterised by its direct and explicit expression between aggressor(s) and victim(s), and being aversive; and
(b) another commonly referred to as isolation, more indirect, implicit, and covert, hardly identifiable by third parties and even by the victim themselves, as the aggressors exercise it through omission of actions, inattention, and avoidance towards the victim [18].

Due to the nature of the most widely used instruments in these studies—sociometric (peer acceptance or rejection) or psychometric (self-report scales on behavioural manifestations)—the theory regarding the existence of two forms of peer social exclusion, one overt and one subtle, still had unclear areas. While overt forms were considered intentional and clearly evidenced, subtle forms were being underestimated, as they did not take into account the feelings of victimisation experienced by children subjected to avoidant and indirect exclusion. Recently, the theory of overt and subtle peer social exclusion has been strengthened by research that has designed and validated a self-report instrument sensitive to the feelings of victimisation generated by both the more direct and evident forms, as well as the more indirect and covert ones [12,13,19].

Recent research has suggested that being in good physical fitness may serve as protective factors against exclusion during the adolescence [13,19,20]. Several studies have suggested that children and adolescents with higher levels of physical fitness may be less likely to experience social exclusion or victimization [20,21,22], while children with poor motor performance experience higher levels of peer rejection in both educational settings [23]. Good physical fitness may contribute to better mental health outcomes in adolescents by boosting their physical self-concept and self-esteem [24]. Furthermore, physically fit individuals are perceived as more competent, attractive, or socially adept, which may reduce their vulnerability to exclusion and peer rejection [25].

Another area that has garnered increasing attention is the relationship between physical activity (PA) and social exclusion. Engaging in PA not only improves physical health but also enhances psychological and social well-being, particularly among children and adolescents [26,27,28]. Psychologically, PA has been linked to reduced symptoms of anxiety and depression, improved mood, and enhanced emotional regulation [29]. Socially, PA provides a structured environment for children and adolescents to engage with their peers, build friendships, and develop teamwork and communication skills [30]. Studies have shown that regular participation in PA can promote social skills, increase self-confidence and self-concept, and provide opportunities for positive peer interactions [24,31,32]. Furthermore, PA may serve as a context where children and adolescents can develop resilience and coping mechanisms that help them navigate social challenges, including exclusion [19,33]. Moreover, participating in PA can help children and adolescents build social networks, which can serve as protective factors against exclusion [34].

However, not all forms of PA may have the same protective effects. The type of PA, the specific characteristics of the activity, the context and the social dynamics of the group can all influence whether PA serves as a protective factor against social exclusion [27,30]. Different types of PA, such as individual versus team sports, contact versus non-contact sports, and activities with or without opposition, may offer varying degrees of social and psychological benefits [34]. Team sports, for example, are often associated with higher levels of social interaction, cooperation, and a sense of belonging, which can mitigate feelings of social exclusion [35]. In contrast, individual sports may provide opportunities for self-reflection and personal growth, which can enhance self-esteem and resilience [36]. Contact sports, for instance, may carry a higher risk of conflict or aggression, which could exacerbate social tensions and lead to exclusion, but may also have a positive impact on bullying prevention [37,38]. On the other hand, non-contact sports or activities that emphasize cooperation over competition may provide a safer and more inclusive environment for participants [32].

The design of effective educational interventions to prevent social exclusion and promote inclusion among adolescents requires further research [27]. While there is growing evidence supporting the protective role of PA against social exclusion, several gaps remain in the literature. For instance, more research is needed to determine which types of PA are most effective in reducing exclusion. In light of recent scientific advances that point to two possible forms of social exclusion-related victimisation among adolescents—overt and subtle, with distinct characteristics [12,13]—it may be strategically valuable to gain insight into which types of PA are most effective in reducing each of these forms. Therefore, the objectives of this manuscript were to analyse whether participation in PA and good physical fitness serve as protective factors against overt social exclusion and subtle social exclusion in adolescents aged 12 to 18 years, and to identify which types of PA are most effective in mitigating each of these forms of exclusion. Based on the objectives of this study, the following hypotheses are proposed: (1) higher physical fitness and regular participation in PA will be associated with lower levels of both overt and subtle social exclusion; (2) competitive, team-based PA with physical interaction (e.g., contact sports) will be more effective in reducing both forms of exclusion compared to non-contact activities [18]; (3) physical fitness will protect against overt exclusion.

## 2. Materials and Methods

### 2.1. Participants

A total of 876 participants, aged between 12 and 19 years (M = 14.91; SD = 1.71 years), took part in the study. The participants were from four educational institutions in Andalusia, Spain. The sample included 426 girls, representing approximately 48.7% of the total. The selection was made on a convenience basis, considering the accessibility of the schools and the willingness of the students to participate in the research. The participants were recruited from educational centers. In the first phase, physical tests were carried out. Students who had any illness, medical condition, or contraindication were excluded from this stage. Those who completed the physical tests proceeded to complete questionnaires designed to gather additional relevant information for the study

### 2.2. Procedure

Descriptive cross-sectional study was conducted. Prior to its implementation, approval was obtained from the educational institutions, along with informed consent from the participants’ families. The project was approved by the Research Ethics Committee of Córdoba, adhering to the ethical principles outlined in the Declaration of Helsinki. Participants, who belonged to a middle socioeconomic level, were informed about the study’s objective and assured that their participation would be anonymous, confidential, and voluntary, with the option to withdraw at any time.

### 2.3. Instruments

#### 2.3.1. Anthropometric Data

Anthropometric data were collected on the first day of assessment, with adolescents being measured barefoot and wearing light clothing. Weight was measured in kilograms (kg) using a Tanita BF 350 scale (Tanita Corporation, Tokyo, Japan) which provides an accuracy of 0.1 kg. Height measurements were taken with a SECA stadiometer (SECA GmbH & Co. KG, Hamburg, Germany), known for its precision, with an accuracy of 0.1 cm. In addition to these measurements, the body mass index (BMI) was calculated for each participant using the formula: BMI = weight (kg)/height (m^2^). BMI was categorized according to the following scale: normal weight (18.5–24.9 kg/m^2^), overweight (25.0–29.9 kg/m^2^), obesity class I (30.0–34.9 kg/m^2^), and obesity class II (35.0–39.9 kg/m^2^).

#### 2.3.2. Physical Activity

The levels of physical activity (PA) of the participants were measured using two questions, derived from the initial question of the PAQ-A questionnaire [39], with some modifications as made in previous studies [24]:

Question 1: PA in your free time: Have you engaged in any physical activity in the last 7 days (the past week)? If yes, how many days did you engage in it?
Question 2: Do you regularly attend any physical activity or sports classes? Please specify the type of activity and the days of the week.

From the responses to these questions, the number of days spent in free-time PA was determined, as well as the days and type of participation in organised PA undertaken by the students. The participants responded by indicating a number from 0 to 7 based on the number of days they engaged in the activity during the week. In both questions, the days corresponding to Physical Education (PE) classes were not counted.

Free-time PA (Question 1) encompassed both free and organised physical activities. On the other hand, organised PA (Question 2) refers to repetitive activities carried out in a club or organisation and led by an instructor. Based on the responses to question 2, those who indicated they participated in organised physical activity also specified the type of physical activity they engaged in, which led to the creation of a new variable called ‘type of organised PA’ in which activities were categorised according to the groups proposed by [24], based on the categorisation by [40].

#### 2.3.3. Physical Fitness Tests

Some of the of the EUROFIT battery was selected, a widely validated tool for physical fitness assessment, was used over the course of three days in this study [41]. Recognized and considered a standard in measuring the physical fitness of adolescents, this battery is reliable both in research and educational practices [42]. In this study, we analyzed the selected fitness tests separately in order to identify which components of physical fitness are more sensitive in their relationship with social exclusion. Additionally, the tests included in the battery are well known to the students, as they are part of their regular educational assessments [43]. By providing a comprehensive evaluation of various aspects of physical fitness, the battery allows researchers to gain valuable insights into adolescents’ overall physical capabilities. The description of the tests is as follows:

(a) 30-S Sit-Up Test: Assesses core muscular endurance. Participants perform as many sit-ups as possible within 30 s, ensuring their elbows touch their knees in each repetition. A Casio HS-80TW stopwatch (Casio Computer Co., Tokyo, Japan) was used to measure time and record the number of correctly executed sit-ups.
(b) Sit-and-Reach Flexibility Test: Measures hip joint mobility. Participants, seated with legs fully extended, reach forward as far as possible with their hands along a ruler attached to a standardized box. A fixed reference point of 15 cm is used to standardize the measurement.
(c) Horizontal Jump Test: Evaluates explosive lower limb strength. From a static position, participants perform a maximum-distance jump. The distance is measured in centimeters using an adhesive tape on the ground. The best of two attempts is recorded.
(d) Aerobic Endurance Test (20 m Shuttle Run Test, SRT): Assesses maximal aerobic capacity. Participants run 20 m back and forth, following auditory signals that progressively increase in speed. They continue until they can no longer maintain the pace. The time is recorded in minutes and seconds.
(e) Handgrip Strength Test: Measures upper limb strength using a TAKEY TKK 5110 dynamometer (Takei Scientific Instruments Co., Niigata, Japan). Participants apply continuous pressure for 2 s. Each hand is tested twice, and the best result, measured in kilograms (with 0.1 kg precision), is recorded.

To avoid interference between tests and ensure the accuracy of the measurements, a testing sequence was established across different days. This approach allowed participants to have enough recovery time between each test.

The evaluation schedule was as follows:

- Day 1: Sit-and-reach flexibility test and abdominal test.
- Day 2: Aerobic endurance test (20 m shuttle run).
- Day 3: Handgrip strength and horizontal jump tests.

This strategic planning follows best practices in physical fitness assessment, ensuring consistent measurements and avoiding performance variations caused by fatigue.

#### 2.3.4. Social Exclusion

To assess levels of social exclusion, the questionnaire used by González-Delgado et al. [12] was administered. This instrument evaluated social exclusion by considering sociodemographic factors, such as educational institution, academic year, and sex, as well as experiences of exclusion within the participants’ school, classroom, peer group, and friendships. The questionnaire comprised two dimensions, each consisting of five items, rated on a four-point Likert scale, ranging from “strongly disagree” (1) to “strongly agree” (4). The internal consistency values were found to be adequate, with α = 0.906 for manifest exclusion and α = 0.896 for subtle exclusion.

### 2.4. Statistical Analysis

Descriptive data were presented as means and standard deviations (SD). To explore the relationships between age, BMI (Body Mass Index), physical fitness levels, and physical activity in the dimensions of subtle exclusion and manifest exclusion, Spearman’s correlation test was used. To compare the levels of social exclusion and manifest exclusion based on the type of organized physical activity practiced, a one-way Analysis of Variance (ANOVA) was applied. Since multiple comparisons between groups were made, the Bonferroni correction was implemented to control the risk of Type I error. Additionally, stepwise regression analyses were performed, considering physical fitness levels, physical activity, BMI, and age as independent variables, and subtle exclusion and manifest exclusion as dependent variables. Statistical significance was set at *p* < 0.05, and the data analysis was carried out using the Statistical Package for the Social Sciences (SPSS) version 29.

## 3. Results

Table 1 presents the correlations between age, BMI, physical fitness, and physical activity participation with both manifest and subtle exclusion. A positive correlation was found between BMI and age with subtle exclusion. A negative relationship was observed between the sit-up test and subtle exclusion. Regarding freely practised and organised physical activity, both variables showed a negative correlation with both manifest and subtle exclusion.

Table 2 examines the relationship between both forms of exclusion and participation in organised physical activity, considering game logic and participant interaction. Engaging in team-based physical activity in a competitive setting, where participant contact and opposition actions are involved, is associated with lower levels of both manifest and subtle exclusion compared to those who do not engage in physical activity. No significant differences were found for individuals who participate in individual, non-competitive, non-contact, and non-oppositional physical activities compared to those who do not practise physical activity.

Table 3 presents a stepwise regression analysis examining how various independent variables—gender, age, BMI, sit-up, sit-and-reach, horizontal jump, shuttle run test, handgrip strength, and organised physical activity—predict the two types of exclusion: manifest and subtle. The analysis was conducted using two separate models.

In the manifest exclusion model, the analysis was carried out in four steps. In the first step, the shuttle run test emerged as a negative predictor, explaining 2% of the variance. In the second step, age was added as a positive predictor, increasing the explained variance to 2.6%. In the third step, sit-up was included as a negative predictor, raising the explained variance to 3.2%. Finally, in the fourth step, gender was introduced as a negative predictor, reaching a total explained variance of 3.8%.

Regarding the subtle exclusion model, a total of three steps were included. In the first step, aerobic endurance test performance was again identified as a negative predictor, explaining 2.6% of the variance. Subsequently, in the second step, age was added as a positive predictor, increasing the explained variance to 4.8%. Finally, in the third step, participation in organised physical activity was incorporated as a negative predictor, achieving a total explained variance of 5.5%.

## 4. Discussion

The objectives of this manuscript were to analyze whether participation in PA and having good physical fitness serve as protective factors against social exclusion in children and adolescents aged 12–19, and to identify which types of PA are most effective in mitigating exclusion. The forms of exclusion among peers in adolescents range from direct manifest exclusion to more indirect or subtle forms of exclusion, and this phenomenon has been recognized as a public health problem [9,10]. Knowing which variables work as a protective factor against exclusion during adolescence could contribute to developing more targeted and effective strategies.

### 4.1. Predictors of Manifest and Subtle Exclusion

In the present study, we found that physical fitness and the practice of free or organized PA could be negative predictors of manifest exclusion. In addition, the sit-up test performance was found to be a negative predictor, while BMI was positively associated with subtle exclusion. No relationship was found between the age, flexibility or jump, and the exclusion. In relation to the results showing that more significant subtle exclusion is found in adolescents with higher BMI, these are in line with some previous studies that showed a negative relationship between peer rejection and body size [44,45]. Furthermore, it has been stated that three out of four students have witnessed how their overweight or obese peers have been suffering from manifest or subtle exclusion [46].

Furthermore, the results of this study also showed a negative relationship between the performance in the sit-up test with subtle exclusion and the shuttle run test with both forms of exclusion. Therefore, it seems that adolescents with higher levels of physical fitness may be less likely to experience exclusion. These findings are consistent with several previous research that have suggested a negative relationship between physical fitness and peer rejection and exclusion [20,23,44]. For example, ref. [20], in a systematic review of children and adolescents, found that self-perceived levels of physical abilities and physical condition were negatively related to subtle exclusion victimization. Similarly, ref. [23] concluded that children with poor motor performance experienced higher levels of peer rejection in both play and classroom settings. Furthermore, these findings align with recent research indicating that high levels of physical fitness may contribute to better mental health outcomes in adolescents by boosting their physical self-concept and self-esteem [24]. This relationship between physical fitness and exclusion may stem from the perception that physically fit individuals are more competent, attractive and socially skilled, which reduces their susceptibility to exclusion [25].

Regarding PA practised freely and in an organized manner, in the present study, a negative correlation was shown in both variables for manifest and subtle exclusion. These findings could support that adolescents who regularly engage in PA experience less victimization than those who are not physically active are consistent with those of the majority of studies [21,47,48]. It has been stated that PA is associated with mental health in young people [26], and this can lead to increased physical self-concept, self-esteem and social support, protecting from exclusion [18,49,50]. Furthermore, it has been demonstrated that physically active adolescents have greater physical fitness [23,51], and both PA practice and physical fitness seem to be negative predictors of manifest and subtle exclusion.

Therefore, the results of this research seem to indicate that exclusion could be negatively associated with physical fitness (sit-up test and shuttle run test), physical appearance (BMI) and PA practice. Flexibility, jump and manual dynamometry were the only performed tests that did not appear to be related to manifest or subtle exclusion in the present study. Similarly, ref. [13] found that exclusion is negatively related to ability, physical condition, and attractiveness, but does not affect strength. These results could broaden the understanding of how different elements influence feelings of manifest and subtle exclusion in adolescents.

### 4.2. Exclusion Based on the Characteristics of PA

Not only the practice of PA is associated with exclusion in adolescents, but the type of performed PA may also play a role in exclusion. It seems that not all forms of PA may have the same protective effects, and some authors have indicated that differences may arise based on the qualitative (type) and quantitative (duration) characteristics of the PA practised [27,52], although this is not totally established yet. According to our results, performing competitive team PA, in which there is contact between participants and there are opposition actions, could be associated with lower levels of manifest and subtle exclusion compared to those who do not practice PA. However, no significant differences were found between those who practice individual, non-competitive, non-contact and non-opposition physical activities and those who do not practice PA.

The role each type of PA plays in exclusion could be due to the specific physical and psychosocial demands of each type of sport. According to the physical characteristics, team competitive sports contribute to significant gains in both upper and lower muscle strength [53], while individuals involved in individual sports typically show superior explosive strength, sprinting capabilities, and skills in acceleration and deceleration [54]. Moreover, previous research has observed that adolescents with lower physical fitness or body size are often excluded from competitive team sports [45,55]. The different demands of each type of sport [56] could explain the results observed in our study because, as we have previously stated, physical condition and exclusion are closely related. Furthermore, if we focus on social development, team sports are linked to increased social interaction and typically involve collaboration, communication and mutual support [35]. This allows children and adolescents to form social networks and increases their sense of belonging, which can act as a protective factor against exclusion [34,35].

In addition to competitive team sports with opposition, the result of the present study also showed that practising contact sports could be a protecting factor from exclusion. Contradictorily, it has been previously stated that contact sports may present a greater risk of conflict or aggression, potentially increasing social tensions [37], and that non-contact sports emphasize cooperation and participants showed significantly higher levels of behaviour and self-concept [32,52]. Even though, there are studies that, consistently with our results, show that contact sports have a positive impact on bullying prevention and related factors [38]. In addition, adolescents who practice contact sports have high levels of muscular strength and greater self-concept [57] compared to adolescents who practice other sport types, both key factors in exclusion protection.

Therefore, these results seem to indicate that not all forms of PA may have the same protective effect against exclusion, and PA characteristics can influence whether they serve as a protective factor. Based on our findings, performing competitive team PA, in which there is contact between participants and opposition actions, seems to be associated with lower levels of manifest and subtle exclusion.

### 4.3. Regression Analysis Predicting the Two Types of Exclusion

The regression analysis performed in the present study indicates that manifest exclusion is significantly influenced by four key predictors (3.8%). First, the shuttle run test, an indicator of aerobic fitness, exhibits a negative association with manifest exclusion, highlighting the potential interplay between physical fitness and manifest exclusion in adolescents. Second, age is positively associated with manifest exclusion, implying that as adolescents grow, they may experience increasing levels of manifest exclusion. Previous research has confirmed that age is a determining factor in peer victimization and bullying behaviour [24,58]. Third, abdominal strength also shows a protective effect. The appearance of shuttle run test and abdominal strength suggests, once again, that adolescents with higher physical fitness are less likely to be manifestly excluded [20,23], potentially due to enhanced self-concept, self-esteem [24] and the perception of being more competent and attractive [25] derived from physical fitness. Lastly, the role of sex emerges as a significant factor, with males being less likely to be manifestly excluded compared to their female peers. This could be because girls appear to be more vulnerable regarding their self-perception of physical attractiveness [59], and the self-perceived level of physical attractiveness serve as a negative statistical predictor for exclusion [13]. Gender differences in exclusion behaviours emphasize the importance of developing tailored interventions that address this issue.

For subtle exclusion, the analysis seems to reveal a similar, yet distinct pattern influenced by three key predictors (5.5%). The shuttle run test remains a significant predictor, with higher aerobic fitness correlating with lower levels of subtle exclusion. This finding reinforces the idea that physical fitness may buffer against various forms of adverse social experiences [20,23]. Additionally, age again is positively related to subtle exclusion, suggesting that subtle exclusionary practices increase as adolescents grow. Another notable predictor for subtle exclusion is participation in organized PA. Engagement in structured sports or PA appears to mitigate subtle exclusion, probably because adolescents who participate in organized PA have greater physical fitness [51], increased physical self-concept and self-esteem [50] and suffer less victimization than those who are not physically active [19,48].

From a practical standpoint, these results could suggest that interventions focusing on improving physical fitness and promoting regular involvement in organized PA could be promising strategies for reducing both manifest and subtle exclusion. Despite these insightful findings, it is important to note that the regression models account for a relatively modest proportion of the variance in exclusion (3.8% for manifest and 5.5% for subtle). This indicates that there are likely additional unmeasured factors that contribute to exclusion, and future research should integrate these additional variables to provide a deeper understanding of the mechanisms underlying both manifest and subtle exclusion among adolescents.

## 5. Conclusions

This study has certain limitations that should be acknowledged. The primary limitation of this study is based on the nature of self-reporting by participants, which could present some response bias. Additionally, the sample is not randomized and does not reflect the full diversity of a specific geographic region. Finally, the last limitation could be the lack of a sex-based comparison across different PA and sport types. Future investigations could focus on examining it and exploring sex differences in the predictors of manifest and subtle exclusion in adolescents.

In conclusion, based on the findings outlined in this manuscript, it could be stated that physical fitness (sit-up test and shuttle run test), physical appearance (BMI) and PA practice may be a protective factor against exclusion. Furthermore, according to our results, performing competitive team PA, in which there is contact between participants and there are opposition actions, may be associated with lower levels of manifest and subtle exclusion compared to those who do not practice PA. These conclusions suggest the need to design more targeted and effective strategies for promoting inclusion and well-being in youth populations, aiming to reduce both manifest and subtle forms of social exclusion.

## Figures and Tables

**Table 1:** Spearman correlations between the different variables, manifest exclusion and subtle exclusion.

Variable (n)	Manifest Exclusion	Subtle Exclusion
Age (876)	0.039	0.076 *
BMI (838)	0.058	0.073 *
Sit-up (826)	−0.063	−0.07 *
Sit-and-reach (846)	0.026	0.009
Horizontal jump (825)	−0.072	−0.069
Shuttle Run Test (821)	−0.144 **	−0.154 **
Handgrip (839)	0.017	0.027
Free physical activity (867)	−0.072 *	−0.112 **
Organised physical activity (869)	−0.132 **	−0.159 **

Note. *p*-values are indicated according to *p* < 0.01 ** *p* < 0.05 *.

**Table 2:** Manifest exclusion and subtle based on the characteristics of organised physical activity, grounded in game logic and participant interactions.

	Manifest Exclusion				Subtle Exclusion		
Subgroup	N	Inter Gr. (*p*)	M ± SD	Intra Gr. (*p*)	Inter Gr. (*p*)	M ± SD	Intra Gr. (*p*)
Competition							
0. No practice	395	0.007	1.31 ± 0.50	2 *	<0.001	1.54 ± 0.65	2 ***
1. No competition	205		1.22 ± 0.46			1.46 ± 0.60	
2. Yes competition	237		1.20 ± 0.41	0 *		1.32 ± 0.53	0 ***
Individual-Team							
0. No practice	395	0.005	1.31 ± 0.50	2 **	<0.001	1.54 ± 0.65	2 ***
1. Individual	91		1.25 ± 0.51			1.44 ± 0.54	
2. Team	351		1.20 ± 0.41	0 **		1.37 ± 0.57	0 ***
Contact							
0. No practice	395	0.002	1.31 ± 0.50	2 **	<0.001	1.54 ± 0.65	2 ***
1. No contact	215		1.25 ± 0.47			1.45 ± 0.60	
2. Yes contact	227		1.18 ± 0.39	0 **		1.33 ± 0.53	0 ***
Opposition							
0. No practice	395	0.007	1.31 ± 0.50	2 *	<0.001	1.54 ± 0.65	2 ***
1. No opposition	156		1.22 ± 0.46			1.44 ± 0.58	
2. Yes opposition	286		1.21 ± 0.42	0 *		1.36 ± 0.56	0 ***

Note. The inter-group differences compare the three groups, while the intra-group differences are analysed at the post-hoc level. *M* = Mean; (*SD*) = Standard Deviation. Established level of significance: *p* < 0.05 *; *p* < 0.01 **; *p* < 0.001 ***.

**Table 3:** Stepwise regression analysis of age, gender, BMI, sit-up, sit-and-reach, horizontal jump, shuttle run test, handgrip, organised physical activity as predictors of manifest and subtle exclusion.

Manifest Exclusion			Subtle Exclusion		
Variable	*β*	*t*	Variable	*β*	*t*
	Step 1 R^2^ = 0.020			Step 1 R^2^ = 0.026	
Shuttle Run Test	−0.141 ***	−3.877	Shuttle Run Test	−0.161 ***	−4.440
	Step 2 ΔR^2^ = 0.006			Step 2 ΔR^2^ = 0.022	
Shuttle Run Test	−0.161 ***	−4.281	Shuttle Run Test	−0.199 ***	−5.365
Age	0.078 *	2.076	Age	0.152 ***	4.087
	Step 3			Step 3	
	ΔR^2^ = 0.006			ΔR^2^ = 0.007	
Shuttle Run Test	−0.120 **	−2.854	Shuttle Run Test	−0.170 ***	−4.349
Age	0.092 *	2.424	Age	0.147 ***	3.963
Sit-up	−0.089 *	−2.108	Organised physical activity	−0.089 *	−2.367
	Step 3 ΔR^2^ = 0.006				
		
Shuttle Run Test	−0.147 ***	−3.354			
Age	0.100 **	2.632			
Sit-up	−0.111 *	−2.559			
Gender (male)	−0.086 *	−2.097			

Note. *p*-values are indicated according to *p* < 0.001 ***; *p* < 0.01 **; *p* < 0.05 *.

## Data Availability

The data supporting the findings of this study can be obtained from the corresponding author upon reasonable request. However, they are not publicly accessible to protect the privacy of the adolescent participants.
